# Quantal amplitude at the cone ribbon synapse can be adjusted by changes in cytosolic glutamate

**Published:** 2011-04-12

**Authors:** Theodore M. Bartoletti, Wallace B. Thoreson

**Affiliations:** 1 Department of Ophthalmology and Visual Sciences, University of Nebraska Medical Center, Omaha, NE; 2 Department of Pharmacology and Experimental Neuroscience, University of Nebraska Medical Center, Omaha, NE

## Abstract

**Purpose:**

Vision is encoded at photoreceptor synapses by the number of released vesicles and size of the post-synaptic response. We hypothesized that elevating cytosolic glutamate could enhance quantal size by increasing glutamate in vesicles.

**Methods:**

We introduced glutamate (10–40 mM) into cone terminals through a patch pipette and recorded excitatory post-synaptic currents (EPSCs) from horizontal or OFF bipolar cells in the *Ambystoma tigrinum* retinal slice preparation.

**Results:**

Elevating cytosolic glutamate in cone terminals enhanced EPSCs as well as quantal miniature EPSCs (mEPSCs). Enhancement was prevented by inhibiting vesicular glutamate transport with 1S,3R-1-aminocyclopentane-1,3-dicarboxylate in the patch pipette. A low affinity glutamate receptor antagonist, γD-glutamylglycine (1 mM), less effectively inhibited EPSCs evoked from cones loaded with glutamate than control cones indicating that release from cones with supplemental glutamate produced higher glutamate levels in the synaptic cleft. Raising presynaptic glutamate did not alter exocytotic capacitance responses and exocytosis was observed after inhibiting glutamate loading with the vesicular ATPase inhibitor, concanamycin A, suggesting that release capability is not restricted by low vesicular glutamate levels. Variance-mean analysis of currents evoked by flash photolysis of caged glutamate indicated that horizontal cell AMPA receptors have a single channel conductance of 10.1 pS suggesting that ~8.7 GluRs contribute to each mEPSC.

**Conclusions:**

Quantal amplitude at the cone ribbon synapse is capable of adjustment by changes in cytosolic glutamate levels. The small number of channels contributing to each mEPSC suggests that stochastic variability in channel opening could be an important source of quantal variability.

## Introduction

The quantal hypothesis of Fatt, del Castillo, and Katz [1,2] postulated that the postsynaptic response is constructed from a sum of quantal synaptic responses, each reflecting the fusion of an individual synaptic vesicle. The postsynaptic response is thus a product of the number of quanta (N), the probability that quanta will be released (P), and the size of individual quanta (Q). These quantal parameters have been measured at several synapses, including the neuromuscular junction, calyx of Held, mossy fiber synapse in the hippocampus, retinal bipolar cell ribbon synapse, and cone photoreceptor ribbon synapse [1-7]. It is often assumed that vesicles are maximally filled with glutamate and quantal amplitude is a fixed parameter. However, amperometric measurements in chromaffin cells have demonstrated variation in catecholamine concentration among dense core vesicles [8]. Additionally, elevating cytosolic L-glutamate in the presynaptic terminal potentiates individual quanta at the calyx of Held, suggesting that individual vesicles are not always fully loaded with glutamate [9]. Adjustments in quantal size by changes in glutamate transporter expression or activity can provide mechanisms for synaptic plasticity [10-12]. In addition, differences in the glutamate concentration among vesicles can be a major source of quantal variability [11].

Cone light responses are encoded by changes in the rate of vesicle release at ribbon synapses. The ribbon is a plate-like protein structure that tethers vesicles near release sites, but its role in release remains unclear [13]. Maintaining consistency in quantal size would ensure more consistent and predictable synaptic output. We therefore asked whether quantal size at the photoreceptor ribbon synapse can be altered by changes in cytosolic glutamate and whether the ribbon reduces postsynaptic variability by restricting release to vesicles that are fully loaded with glutamate. Our results showed that increasing cytosolic glutamate levels at the cone ribbon synapse enhanced postsynaptic responses by increasing vesicular glutamate levels. Elevation of vesicular glutamate levels did not enhance release, and exocytosis persisted after blocking vesicular glutamate loading, arguing against an internal checkpoint mechanism. Using nonstationary fluctuation analysis techniques to determine the single-channel conductance for α-amino-3-hydroxy-5-methyl-4-isoxazolepropionic acid (AMPA) receptor currents in horizontal cells, we found that <10 receptor openings contributed to each miniature excitatory postsynaptic current (mEPSC). Together, these results suggest that quantal amplitude at the cone synapse can be adjusted by physiologic activity, that variations in vesicular glutamate levels can be an important source of quantal variability, and that quantal variability may be enhanced by stochastic variability in the number of open channels contributing to each mEPSC.

## Methods

### Retinal slice preparation

Aquatic tiger salamanders (*Ambystoma tigrinum*; Kons Scientific, Germantown, WI‎ or Charles Sullivan Co., Nashville, TN) 18–25 cm in length were handled humanely according to protocols approved by the Institutional Animal Care and Use Committee at the University of Nebraska Medical Center. Salamanders were decapitated with heavy shears, and the brain and spinal cord were then rapidly pithed.

Animals were kept on a 12 h:12 h light-dark cycle and sacrificed 1–2 h after the beginning of subjective night. The electrophysiological techniques and retinal slice preparation were performed according to previously published methods [7,14]. Briefly, after the anterior segment of the eye was removed, the resulting eyecup was cut into thirds, and a section was placed vitreal-side down on a piece of filter paper (2×5 mm, Type AAWP, 0.8 μm pores; Millipore, Bedford, MA). After the retina adhered to the filter paper, the retina was isolated under chilled amphibian superfusate. The retina and filter paper were cut into 125 μm slices using a razor blade (#121–6; Ted Pella Inc., Redding, CA) tissue chopper (Stoelting, Wood Dale, IL). Retinal slices were rotated 90° to permit viewing of the retinal layers when placed under a water immersion objective (40×, 0.7 NA or 60×, 1.0 NA) and viewed on an upright fixed-stage microscope (Olympus BHWI or Nikon E600FN, Tokyo, Japan).

Solutions were applied by a single-pass, gravity-feed perfusion system, which delivered superfusate to the slice chamber at a rate of ~1 ml/min. The normal amphibian superfusate contained: 111 mM NaCl, 2.5 mM KCl, 2 mM CaCl_2_, 0.5 mM MgCl_2_, 10 HEPES, and 5 mM glucose (pH 7.8). Use of HEPES as a pH buffer limited the effects of proton feedback [15-17]. For some experiments, 2,3-dihydroxy-6-nitro-7-sulfamoyl-benzo[f]quinoxaline-2,3-dione (NBQX; 1 μM), γ-D-glutamylglycine (γDGG; Tocris Bioscience, Ellisville, MO), or 0.1 mM glutamine was added to the superfusate. The osmolarity was measured with a vapor pressure osmometer (Wescor, Logan, UT) and adjusted, if necessary, to 242±5 mOsm. Solutions were bubbled continuously with 100% O_2_.

### Electrophysiological recording and analysis

Patch pipettes for voltage clamp recording were pulled on a PP-830 vertical puller (Narishige USA, East Meadow, NY) from borosilicate glass pipettes (1.2 mm O.D., 0.9 mm I.D., with internal filament, World Precision Instruments, Sarasota, FL). The resulting electrodes had tips of ~1 μm O.D. with resistance values of 10–15 MΩ. The presynaptic control pipette solution was composed of: 90 mM CsGluconate, 10 mM tetraethylammonium chloride (TEACl), 3.5 mM NaCl, 1 mM CaCl_2_ 1 mM MgCl_2_, 10 mM magnesium ATP (MgATP), 0.5 mM GTP, 5.0 mM EGTA, and 10 mM HEPES (pH 7.2). The presynaptic high-glutamate pipette solution contained: 40 mM CsGlutamate, 50 mM CsGluconate, 10 mM TEACl, 3.5 mM NaCl, 1 mM CaCl_2_, 1 mM MgCl_2_, 10 mM MgATP, 0.5 mM GTP, 5 mM EGTA, and 10 mM HEPES (pH 7.2). For some experiments, we substituted 40 mM alpha ketoglutarate or 40 mM glutamine in place of glutamate. In one set of experiments, 0.5 mM of 1S,3R-1-aminocyclopentane-1,3-dicarboxylate (1S,3R-ACPD), was added to the 40 mM glutamate intracellular solution. Chloride concentrations in the pipette solutions were matched because glutamate uptake shows a bell-shaped dependence on intracellular chloride, with optimal uptake at low millimolar concentrations [12,18]. Postsynaptic pipettes were filled with a solution containing: 48 mM CsGluconate, 42 mM CsCl, 9.4 mM TEACl, 1.9 mM MgCl_2_, 9.4 mM MgATP, 0.5 mM GTP, 5 mM EGTA, and 32.9 mM HEPES (pH 7.2). The osmolarity of pipette solutions was adjusted, if necessary, to ~240 mOsm.

Cones were voltage-clamped simultaneously with adjacent postsynaptic horizontal or OFF bipolar cells using a Multiclamp patch-clamp amplifier (Molecular Devices, Sunnyvale, CA). Cones and horizontal cells were identified by their morphology and response characteristics [19]. Both recording pipettes were positioned with Huxley-Wall micromanipulators (Sutter Instruments, Novato, CA) and visualized through the eyepieces or with a video camera (502H; Watec, Orangeburg, NY) mounted on the microscope.

Cones were voltage-clamped at a steady holding potential of −70 mV between test pulses. Horizontal and OFF bipolar cells were held at −60 mV. Acceptable access resistance for voltage clamp recordings was <60 MΩ. Currents were low pass-filtered at 2 kHz and acquired using a Digidata 1322 interface with pClamp 9.2 software (Molecular Devices).

Quantal mEPSCs in horizontal and bipolar cells were detected and analyzed using Minianalysis 6.0.7 (Synaptosoft, Inc., Decatur, GA) as described previously [20]. Events were initially detected using an amplitude threshold of 1 pA and an area threshold of 1 pC. Each event was then evaluated individually, and if necessary, the preceding baseline period was adjusted in length to improve the amplitude measurement. Double peaks were analyzed using an algorithm within Synaptosoft that extrapolates the exponential decay of the first peak.

For capacitance recordings from cones, pipettes were coated with dental wax to reduce stray capacitance. Cell capacitance and residual pipette capacitance were compensated electronically. Capacitance measurements were made using the “track-in” mode of the Optopatch (Cairn Research, Faversham, UK) patch-clamp amplifier [21,22]. The holding potential was varied sinusoidally (500–600 Hz, 30 mV peak to peak) about a mean value of −70 mV. The amplitude of the cone capacitance response was measured 30 ms after the end of the test step to avoid gating charges and allow time for the phase-angle feedback circuitry to settle.

For experiments with caged glutamate, 4-methoxy-7-nitroindolinyl-L-glutamate (MNI glutamate; 0.5 mM; Tocris Biosciences) was added to the superfusate and photolyzed by flashes of UV light derived from a Xenon arc flash lamp (JML-C2 Flash Lamp System; Rapp Optoelectronic, Hamburg, Germany). In some experiments, a D1 dopamine receptor antagonist, SKF38393 (10 μM), was also applied to uncouple horizontal cells and thereby limit effects of flash photolysis on horizontal cell coupling. Light flashes (17.5 μm diameter) were delivered via a quartz optic fiber through the epifluorescence port of the microscope and centered on the horizontal cell. Nonstationary fluctuation analysis was used to determine single-channel glutamate receptor (GluR) conductance. A series of GluR currents were evoked by UV light flashes. Currents were aligned at their peaks, peak-scaled [23], and binned in 5 ms increments using Minianalysis 6.0.7 (Synaptosoft, Inc.). Peak scaling reduces the effects of run-down but eliminates information about open probability and the number of channels. However, the mean–variance relationship remains parabolic and provides an estimate of single-channel current, *i* [24,25]. The mean and intertrace variance were calculated for each 5 ms bin and then fit with Equation 1:

$${\text{Var(t) = i*I(t) - I(t)}}^{2}\text{ / B + offset,}$$

where i=single-channel current amplitude and n=number of receptors.

Unless otherwise noted, chemicals and reagents were obtained from Sigma-Aldrich (St. Louis, MO). The criterion for statistical significance chosen was p<0.05 and evaluated using GraphPad Prism 4.0 (La Jolla, CA). Variability is reported as ±standard error of the mean (SEM).

## Results

### Elevating cytosolic glutamate in the cone terminal increased the amplitude of excitatory postsynaptic currents

**Figure 1:**
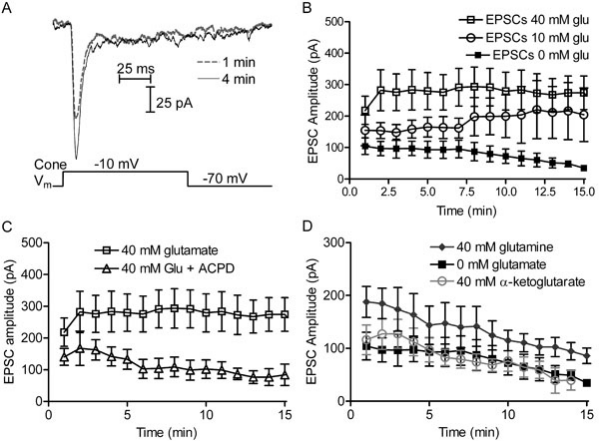
Increasing cytosolic glutamate levels in cone terminals enhanced the amplitude of excitatory postsynaptic currents (EPSCs) recorded from postsynaptic horizontal cells. **A**: Examples of horizontal cell EPSCs recorded the first minute (thin black trace) after obtaining whole-cell configuration, and 8 min later (dashed gray trace) when using a pipette solution containing 40 mM glutamate. EPSCs were evoked by depolarizing the cone from −70 mV to −10 mV for 100 ms. **B**: EPSCs recorded every minute with 40 mM glutamate (open squares, n=8) in the patch pipette are much larger than EPSCs recorded without glutamate (filled squares, n=8) in the patch pipette. Use of 10 mM glutamate produced less enhancement (open circles, n=7). For three of the cell pairs with 10 mM glutamate, measurements were made for only 10 min. **C**: EPSCs recorded with 40 mM glutamate (open squares) were larger than EPSCs with 40 mM glutamate plus 0.5 mM 1S,3R-1-aminocyclopentane-1,3-dicarboxylate (open triangles, n=4), a potent vesicular glutamate transport inhibitor. **D**: Substituting 40 mM alpha ketoglutarate (open gray circles, n=4) for glutamate did not enhance the EPSC amplitude above that of EPSCs recorded without glutamate (filled squares). Substituting 40 mM glutamine (n=8, filled diamonds) for glutamate slightly enhanced the EPSCs. Error bars show SEM.

To determine whether introducing glutamate into the cone terminal through a whole-cell pipette increased the amount of glutamate in synaptic vesicles, we recorded excitatory postsynaptic currents (EPSCs) after dialyzing cone photoreceptors with 40 mM glutamate. Consistent with findings from the calyx of Held [9,26], adding glutamate to the cone pipette solution caused horizontal cell EPSCs to increase over time (Figure 1). To characterize the time course of this enhancement, we recorded EPSCs every minute for 15 min. When 40 mM glutamate was included in the cone pipette solution, the EPSCs grew in amplitude by 81.4±49.2% (n=8) within the first 2 min after patch rupture, and remained much larger than the EPSCs measured in the control cell pairs without added glutamate throughout the 15 min period of the experiment (Figure 1B). In addition to enhancing EPSC amplitude, the addition of cytosolic glutamate appeared to stabilize the EPSCs, slowing the decline in EPSCs that was observed without added glutamate. Use of 10 mM glutamate in the cone pipette solution also augmented the postsynaptic currents, although the effects were not as great as those of 40 mM glutamate (Figure 1B, open circles). To test the possibility that we were simply replacing glutamate lost from retinal slices after their removal from the eye, we included the glutamate precursor, glutamine (0.1 mM), in the bath solution. In the presence of 0.1 mM glutamine, EPSCs grew by 82±35% (n=5) over the first 3 min when 40 mM glutamate was included in the cone pipette, similar to effects without glutamine in the superfusate.

De-amination of glutamate yields alpha-ketoglutarate, which can fuel the Krebs cycle. Mammalian cone terminals possess five mitochondria/active zones, suggesting a high metabolic demand [27]. Salamander cones lack terminal mitochondria, and so there is a rapid rundown of EPSCs in the absence of ATP [28]. Alpha-ketoglutarate derived from the glutamate metabolism has been shown to provide a metabolic alternative to glucose in retinal neurons [29]. We therefore tested the possibility that additional presynaptic glutamate might enhance postsynaptic currents by providing extra metabolic fuel to the cone terminal. However, using 40 mM α-ketoglutarate rather than 40 mM glutamate in the cone pipette solution did not enhance the EPSCs (Figure 1D).

To test whether the enhancement of postsynaptic responses from supplementary presynaptic glutamate requires vesicular loading, we inhibited the vesicular glutamate transporter by including 0.5 mM 1S,3R-ACPD [30], along with 40 mM glutamate, in the cone patch pipette (Figure 1C). In the presence of 1S,3R-ACPD, the EPSCs did not increase over time, suggesting that the enhancing effects of elevated presynaptic glutamate required that glutamate must be loaded into synaptic vesicles by vesicular glutamate transporter activity.

In addition to testing the effects of extracellular glutamine, we also tested the effects of elevating cytosolic glutamine to a concentration of 40 mM in the cone patch-pipette solution. Similar to the addition of 10 mM glutamate, glutamine (40 mM) potentiated EPSCs to levels slightly above those recorded without added glutamine or glutamate (Figure 1D). Thus, glutamate derived metabolically from glutamine might also have enhanced intravesicular glutamate loading. The finding that the enhancement by 40 mM glutamine was less than the enhancement by 40 mM glutamate may be explained by the necessity for enzymatic conversion of glutamine to glutamate.

We tested the possibility that an increase in glutamate release caused by an elevation of cone cytosolic glutamate levels might potentiate postsynaptic AMPA receptors [31]. To test whether elevation of extracellular glutamate could potentiate AMPA receptor currents, we photolytically liberated glutamate from MNI-glutamate (0.5 mM) in the bath while recording from a horizontal or OFF bipolar cell, but not from a cone. Uncaging flashes evoked large inward currents in horizontal cells [32]. When horizontal cells were uncoupled by inclusion of a D1 dopamine receptor antagonist, SKF38393 (10 μM), the currents evoked by the flash photolysis of caged glutamate did not show an enhancement when a second uncaging flash was applied 1 min later (first uncaging flash: 353±88 pA; second flash: 344±91 pA, n=4, p<0.88, paired *t*-test). In OFF bipolar cells, responses to flash photolysis of caged glutamate were not enhanced either (n=4, paired *t*-test, p<0.88).

### Elevated cytosolic glutamate increased miniature excitatory post-synaptic current amplitude

**Figure 2:**
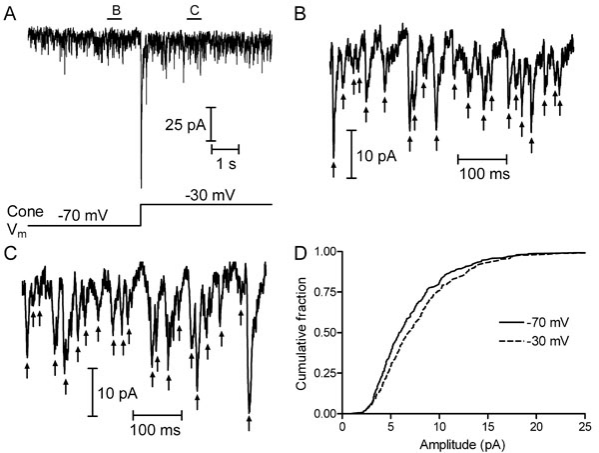
The amplitude of miniature excitatory postsynaptic currents (mEPSCs) increased after dialyzing cones with 40 mM glutamate. **A**: This shows the postsynaptic current recorded from a horizontal cell before and after depolarization of the presynaptic cone to −30 mV. Letters above the trace identify the short segments that are reproduced at a higher timescale in panels **B** and **C**. **B**: Sample of baseline mEPSCs recorded from a light-adapted horizontal cell before stimulation. Individual mEPSCs are indicated by arrows. **C**: Sample of mEPSCs obtained while depolarizing the presynaptic cone to −30 mV. **D**: Cumulative amplitude histograms of mEPSCs recorded during baseline conditions (solid line, n=336 events) and during a step to −30 mV (dashed line, n=360 events) from the cell pair shown in the previous panels. In this example, the mean amplitude increased from 7.11±0.24 pA to 7.91±0.24 pA, following application of the depolarizing step. The frequency of mEPSCs increased from 65.7 to 72.0 Hz.

The ability of 1S,3R-ACPD to block enhancement suggested that elevation of cytosolic glutamate would enhance vesicular loading of glutamate. We therefore examined the effects of supplemental glutamate on individual mEPSCs. Photoreceptors released glutamate continuously at the dark resting membrane potential of –40 mV, and light inhibited this release by hyperpolarizing the photoreceptors. To help distinguish between evoked mEPSCs released from the voltage-clamped cone and spontaneous mEPSCs arising from release from neighboring cones, we applied a saturating background light to inhibit release from neighboring photoreceptors and then stimulated release from a voltage-clamped cone by applying a weak depolarizing step to –30 mV or −40 mV. This mild depolarization produced a small but significant increase in the frequency of mEPSCs recorded in OFF bipolar or horizontal cells (from 59±13 to 63±12 Hz; n=7, p=0.0075, paired *t*-test). When the cone pipette solution was supplemented with 40 mM glutamate, there was a slight but statistically significant increase in the amplitude of mEPSCs evoked by stimulation of the voltage-clamped cone, compared with the amplitude of spontaneous mEPSCs (5.23±0.37 pA to 5.83±0.33 pA; n=9 cell pairs, p=0.0056, paired *t*-test). This increase was most easily seen by a shift in the cumulative amplitude histogram (Figure 2D). The increase in mEPSC amplitude observed following elevation of presynaptic glutamate in the voltage-clamped cone was much smaller than the increase in EPSC amplitude, because most of the mEPSCs were due to spontaneous release by other presynaptic cones and rods, whereas the EPSC reflected release from only the voltage-clamped cone. An increase in mEPSC amplitude during application of a weak depolarizing step was not observed in the control cone/horizontal cell pairs without supplemental glutamate (see Figure 1 from [7]). Moreover, in cones with supplemental glutamate, after killing the cone, there was no increase in amplitude (n=6 cells, p=0.34, paired *t*-test) or release frequency (n=6, p=0.22, paired *t*-test) during the mild depolarization. This supported the hypothesis that increasing cytosolic glutamate levels in the cone terminal augmented the accumulation of glutamate into vesicles, causing an increase in the mEPSC amplitude, which increased the resulting EPSC.

### Elevated cytosolic glutamate increased extracellular glutamate in the synaptic cleft

**Figure 3:**
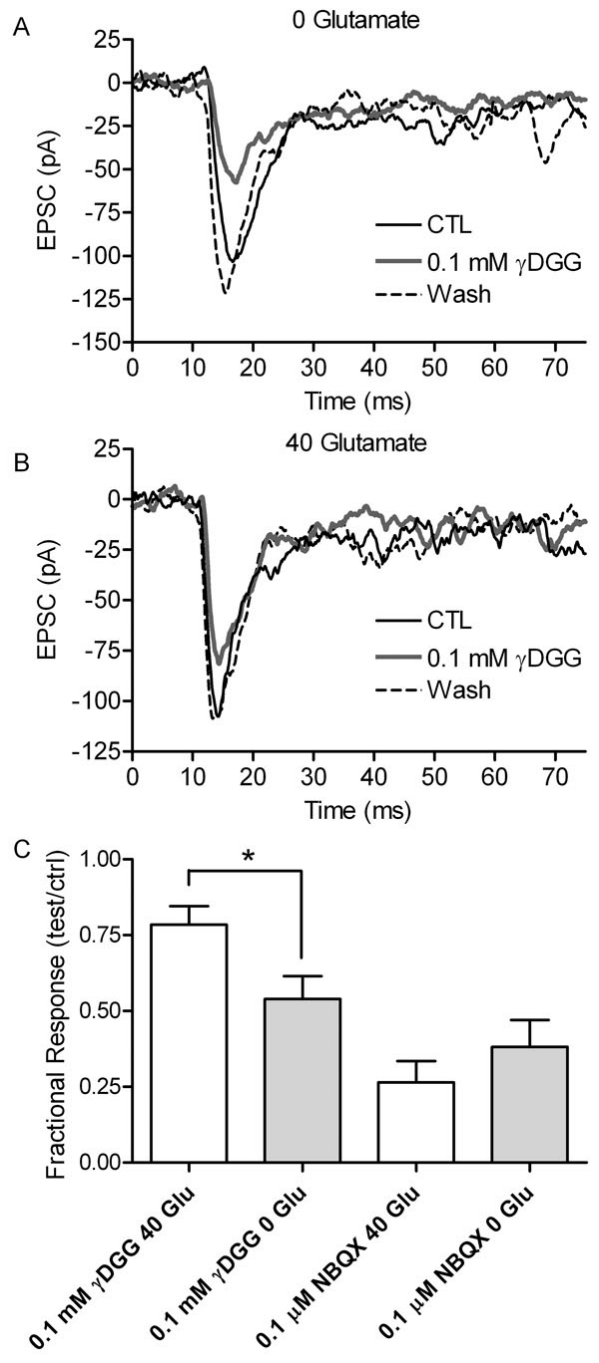
Dialyzing cones with 40 mM glutamate increased the concentration of glutamate within the synaptic cleft. **A**: Example of horizontal cell excitatory postsynaptic currents (EPSCs) evoked by depolarizing stimulation of a presynaptic cone without supplemental glutamate in control conditions (black trace), in the presence of a low-affinity glutamate receptor antagonist γDGG (0.1 mM, gray trace), and following washout (dashed trace). **B**: Example of horizontal cell EPSCs evoked by depolarizing stimulation of a presynaptic cone dialyzed with 40 mM glutamate in control conditions (black trace), during application of γDGG (gray trace), and following washout (dashed trace). **C**: Bar graph showing the fractional inhibition of EPSCs produced by 2.5 min. bath application of γDGG (control, n=9; 40 mM, n=6; p=0.032, unpaired *t*-test) or the high-affinity AMPA antagonist, NBQX (0.1 mM; control, n=8; 40 mM glutamate, n=4; p=0.41, unpaired *t*-test). Error bars show SEM.

Inhibition of postsynaptic responses by low-affinity glutamate antagonists can be used to assess the concentration of synaptic glutamate [26,33]. If elevated cytosolic glutamate levels cause an increase in intravesicular glutamate concentration, then this should result in a higher glutamate concentration in the synaptic cleft. The presence of higher cleft glutamate levels would in turn reduce inhibitory effects of a low-affinity, competitive antagonist [34]. We therefore compared the efficacy of a low-affinity glutamate receptor antagonist, γDGG, in cell pairs with supplemental glutamate in the cone pipette solution and in control pairs without supplemental glutamate. Consistent with higher vesicular glutamate levels, inhibition by γDGG (0.1 mM) was reduced when cones were dialyzed with 40 mM glutamate, compared to recordings without glutamate (Figure 3). In cone-horizontal cell pairs when 40 mM glutamate was added to the cone cytosol, 0.1 mM γDGG inhibited EPSCs by 22±6.1% (n=6). By contrast, in control cells without added glutamate, 0.1 mM γDGG reduced cone-driven EPSCs by 46±7.5% (n=9, p=0.0370, unpaired *t*-test). We observed similar results using a different low-affinity antagonist, kynurenic acid (0.1 mM, data not shown), but focused on γDGG, because kynurenic acid can also act as a competitive inhibitor of the vesicular glutamate transporter [35,36]. In control experiments, inhibition of EPSCs by the high-affinity antagonist NBQX (0.1 μM) did not differ significantly (p=0.4075) when 40 mM glutamate was added to the cone pipette solution (77±9.6% inhibition, n=4) or when glutamate was omitted from the pipette solution (62±8.8% inhibition, n=8; Figure 3).

### Effects of cytosolic glutamate on vesicle release

**Figure 4:**
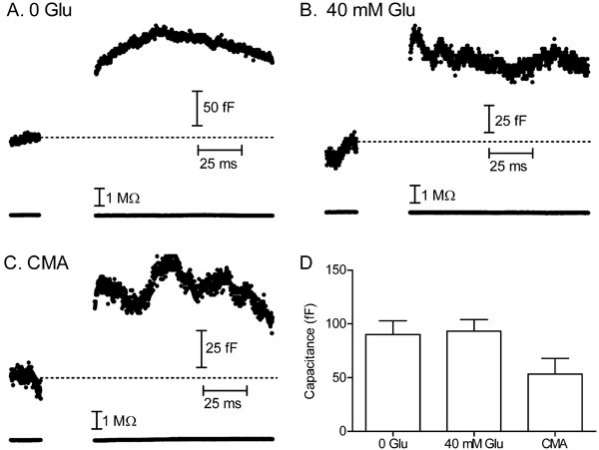
Vesicle fusion was not significantly altered by changes in vesicular glutamate loading. **A**: Exocytotic increase in membrane capacitance (C_m_) evoked by stimulation of a cone lacking supplementary glutamate in the cytosol. Access resistance was not significantly altered (bottom trace). **B**: Exocytotic capacitance increase evoked by stimulation of a cone with 40 mM glutamate added to the pipette solution. **C**: Exocytotic capacitance increase evoked by stimulation of a cone lacking cytosolic glutamate and following pretreatment with the vesicular ATPase inhibitor concanamycin A (CMA, 3.5 μM) for 1 h. **D**: Exocytotic increases in membrane capacitance evoked by depolarizing stimulation of cones (25 ms, −70 to −10 mV) without supplemental glutamate (n=7), with 40 mM glutamate added to the patch pipette (n=6), and after >1 h incubation in concanamycin A with no glutamate in the patch pipette (n=6). Error bars show SEM.

It was possible that the ribbon synapses possessed a checkpoint mechanism that only permitted fusion of vesicles that were fully loaded with glutamate. The addition of cytosolic glutamate might also have expanded the size of individual vesicles [37]. To test these possibilities, we used capacitance techniques to measure the increase in membrane surface area accompanying vesicle fusion evoked by test steps from −70 to −10 mV (25 ms). Capacitance measurements were obtained about five minutes after patch rupture. We found no significant change in the exocytotic increase in membrane capacitance when comparing cones with 40 mM glutamate added to the pipette solution and cones without added glutamate (p=0.55, unpaired *t*-test; Figure 4).

We also tested for a check-point mechanism that limited the release to fully loaded vesicles by blocking glutamate loading with the vesicular ATPase inhibitor, concanamycin A. For these experiments, we pretreated retinal slices with concanamycin A (3.5 μM) for at least 1 h, and used a patch-pipette solution without supplemental glutamate. With ~13 ribbons/cone and a dark rate of release of 20 vesicles/ribbon/s [38,39], there should have been a turnover of nearly 1 million vesicles within 1 h, more than the total number of vesicles in a cone terminal [40]. Consistent with depletion of glutamate from synaptic vesicles, concanamycin A treatment abolished spontaneous mEPSCs and light-evoked currents in horizontal cells (n=3). Although most if not all vesicles were depleted of glutamate, cones remained capable of exocytosis, as evidenced by capacitance measurements. As illustrated in Figure 4, depolarizing test steps (25 ms, −70 to −10 mV) evoked large exocytotic capacitance jumps even after concanamycin A treatment. We excluded experiments in which capacitance jumps were accompanied by significant changes in access resistance. We confirmed that these responses represented true exocytotic capacitance increases by determining whether, unlike calcium-activated conductance changes, they were depressed by applying pairs of pulses separated by 75 ms intervals. Depolarization-evoked capacitance increases that were recorded after concanamycin A treatment averaged 47.7±5.8 fF, which corresponded to the release of ~840 vesicles/cone or ~60 vesicles/ribbon (Figure 4C). Capacitance jumps were smaller than those recorded without the toxin. Although this might have reflected the presence of a check-point mechanism that prevented fusion of a subset of vesicles, it was more likely due to the generally poor health of cones after 1–2 h exposure to concanamycin A. The ability of cones to release vesicles following treatment with concanamycin A suggests that there is not a check-point mechanism that only permits release of fully loaded vesicles.

### Single-channel conductance of horizontal cell glutamate receptors

**Figure 5:**
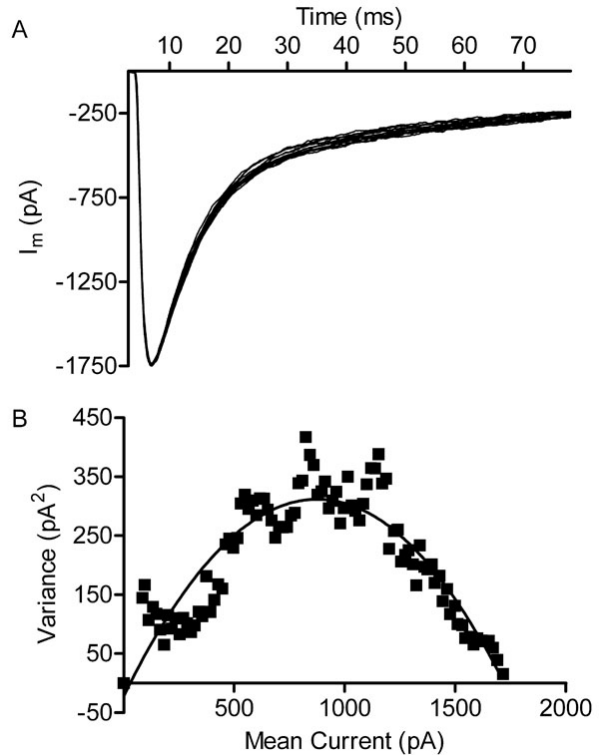
Nonstationary fluctuation analysis of glutamate currents evoked in horizontal cells by flash photolysis of caged glutamate yielded the single-channel glutamate receptor conductance. **A**: Overlay of peak-scaled glutamate receptor membrane currents evoked in a single horizontal cell by flash uncaging of extracellular 0.5 mM MNI-glutamate (n=16). We used a concentration well above the K_d_ for AMPA receptors to eliminate quantal variability and provide a reliable estimate of single-channel conductance. Note the increased variance between traces as the current decays from its peak. **B**: A plot of the variance between traces and mean amplitude of glutamatergic currents. The family of traces shown in A was binned in 5 ms intervals beginning at the peak of the inward current. The mean amplitude and variance between traces was calculated for each 5 ms bin. The resulting variance–mean relationship was fit with a parabolic equation (solid line). In this example, the single-channel current recorded at the holding potential of −60 mV was 0.762 pA or 12.7 pS.

Quantal mEPSCs at the cone synapse were found to average 5.7 pA [14,20], smaller than the amplitude of non-NMDA mEPSCs at many other glutamatergic synapses [e.g., 4,41-43]. This could be due to a large cleft volume, low density of postsynaptic GluRs on horizontal cell dendrites, or unusually low single-channel conductance. To test the latter possibility, we determined the single-channel conductance of GluRs on horizontal cell dendrites by performing nonstationary fluctuation analysis of glutamatergic currents evoked by the flash photolysis of 0.5 mM MNI-glutamate [44]. GluR currents were evoked by a bright UV flash every 45–60 s for up to 30 min. Using MNI-glutamate eliminated the quantal variability observed with depolarization-evoked EPSCs, and so the primary source of variability in the GluR currents arose from stochastic channel openings. The mean and variance between traces of peak-scaled GluR currents (Figure 5A) were calculated in 5 ms intervals, and the mean-variance relationship was fit with a parabola using Equation 1 (Figure 5B; see Methods). The single-channel conductance (n=8 cells, 19±2 measurements per cell) averaged 10.1±1.0 pS (0.66±0.07 pA at −65 mV), similar to the single-channel conductance values found with expressed AMPA receptors and endogenous GluRs in several other preparations [45].

Single-channel conductance measurements allowed us to estimate the number of GluRs that contributed to EPSCs at the cone ribbon synapse. The conductance of an individual quantal mEPSC was 87.7 pS, and so a single-channel conductance of 10.1 pS suggested that ~8.7 GluRs contributed to each mEPSC.

## Discussion

The results of this study showed an increase in EPSC amplitude at cone synapses over time, when cytosolic glutamate in the cone terminal was increased to 40 mM. The enhancement of EPSCs was accompanied by an enhancement of individual mEPSCs, and this was inhibited by blocking vesicular glutamate uptake, suggesting that it resulted from an enhancement of glutamate loading into synaptic vesicles. This conclusion was further supported by the finding that higher cleft-glutamate levels were attained during EPSCs, following the elevation of cytosolic glutamate. We ruled out several alternative possibilities, such as metabolic enhancement, increases in the size or number of vesicle fusion events, or the potentiation of postsynaptic AMPA receptors. Results at the cone synapse were consistent with findings at the calyx of Held, which showed a potentiation of EPSCs after cytosolic glutamate was increased by 10–100 mM [9,26,46].

The concentration of transmitter within synaptic vesicles depends on the concentration of substrate available in the cytoplasm [11]. Consistent with a role for vesicular transport, vesicular glutamate uptake exhibits a K_m_ in the low millimolar range and kinetics similar to those of the enhancing effects of glutamate on EPSCs [47-50]. Vesicular glutamate levels are thought to range from 60 mM to 210 mM [33,51,52], which would necessitate active transport even when cytosolic glutamate levels are elevated to 40 mM. Consistent with this prediction, the enhancement of EPSCs by elevated cytosolic glutamate was blocked by inclusion of the vesicular glutamate transporter antagonist, (1S,3R)-ACPD, in the cytosol. This finding supports the claim that vesicular glutamate transporter activity plays a central role in determining the amplitude of postsynaptic responses. It also suggests that regulation of transporter expression [53] or transporter activity by chloride ions, pH, G proteins, metabolism, or the electrochemical gradient could influence quantal size and synaptic strength [11,12,17,54,55]. Most of the glutamate released as neurotransmitter derives from the precursor glutamine, and our addition of glutamine to the cytosol produced a modest enhancement of EPSCs similar to the effects of exogenous glutamate. This supports the possibility that metabolically driven increases in cytosolic glutamate levels can alter the strength of synaptic signaling [56].

Because the postsynaptic response at cone ribbon synapses is the sum of individual quanta [20], variability in the size of individual quanta will introduce noise into signals transmitted across the synapses and thereby impair the postsynaptic detection of light-evoked changes in release [57]. Like other CNS synapses [4,11,26,58,59], quantal variability at photoreceptor synapses is quite large, with a coefficient of variation of 0.65 [14]. Variability at the cone synapse is not due to poor space clamp [14]. Among other explanations proposed to account for quantal variability are differences in vesicle diameter and intravesicular glutamate concentration [6,11,59-61]. The amount of transmitter released is proportional to vesicle size [62], and the addition of glutamate can increase the diameter of individual synaptic vesicles isolated from rat brain [37]. However, we found that enhancing cytosolic glutamate did not increase the amount of membrane fusion stimulated by synaptic release, suggesting that it did not increase vesicle size. This is consistent with other studies showing greater variability in vesicular glutamate concentration than in vesicle diameter [9-11,26,46,56,63].

Limiting release to fully loaded vesicles could potentially limit any quantal variability that might be introduced by differences in vesicular glutamate concentration. However, capacitance measurements from cones showed that release was unchanged by elevating cytosolic glutamate, and that substantial release occurred even after depleting vesicles of glutamate by treatment with concanamycin A. This is consistent with studies on motor nerve terminals, which used the activity-dependent dye, FM1–43, to show that synaptic vesicles depleted of acetylcholine could still be released [64]. Capacitance measurements cannot eliminate the possibility of a selective increase in kiss-and-run fusion events, but our results are consistent with earlier findings suggesting that the probability of exocytosis is independent of the state of vesicle filling [65].

GluRs on horizontal cell dendrites appear to be formed primarily from the AMPAR subunits GluR2/3 and GluR4 [66-68], although there is immunohistochemical evidence for GluR6/7 kainate receptors in some species [69]. Mean-variance analysis of glutamatergic currents evoked by the flash photolysis of caged glutamate has shown that the single-channel conductance of individual glutamate receptors in horizontal cells of 10.1 pS is similar to the conductance of AMPA receptors in heterologous expression systems and other CNS preparations [45]. The finding that only 8.7 channels contribute to each mEPSC suggests that stochastic variability in the opening of individual channels may contribute to quantal variability at the synapse, since the failure of only a single channel to open would reduce the mEPSC by 11%.

The initial transient component of the EPSC evoked by strong depolarizing stimulation of a cone reflects the release of the entire readily releasable pool, which averages ~20 vesicles per ribbon [7]. Because single quantal mEPSCs act independently from one another at the cone synapse [20], this suggests that a total of ~180 receptors should be activated at each ribbon by the release of the entire readily releasable pool. Freeze-fracture electron micrographs of primate retina show 100 nm-wide particle arrays on horizontal cell dendrites adjacent to the cone synaptic ridge [70], and salamander cone ribbons have a base length of 150–350 nm [39]. These data suggest that AMPA receptors are likely to be confined within a membrane area of 0.015–0.035 μm^2^ on the horizontal cell dendrite. Packing 180 receptors into this area yields a high density of AMPA receptors, exceeding 5,000 per μm^2^ [43,62,71]. The small size of mEPSCs at the cone ribbon synapse does not therefore appear to be due to an unusually small single-channel AMPAR conductance or to low receptor density. It is more likely due to the large diffusional volume of the cleft within the invaginating synapse.

The enhancement of quantal mEPSCs by the addition of cytosolic glutamate supports other findings indicating that postsynaptic receptors are not normally saturated by glutamate release at the photoreceptor synapse [14,20]. AMPA receptors at other ribbon and nonribbon CNS synapses are not saturated during synaptic glutamate release either [9,34,43,72,73]. The concentration of a low-affinity antagonist, which is needed to antagonize postsynaptic responses, provides an estimate of the cleft glutamate concentration [33]. Although receptors do not appear to reach saturation at the cone synapse, the inhibitory effects of γDGG at the cone synapse suggest that glutamate attains a concentration of ≥100 μM, nearing the top of the AMPA receptor dose/response curve [74].

Postsynaptic responses are a product of release probability, releasable pool size and quantal amplitude. Processes regulating release probability and pool size can be regulated by many different mechanisms [75-77]. The present findings indicate that, like release probability and vesicle pool size, quantal amplitude is not a fixed parameter at the cone ribbon synapse, but it is capable of adjustment by physiologic activity.
